# Effects of chronic low‐dose aspirin treatment on tumor prevention in three mouse models of intestinal tumorigenesis

**DOI:** 10.1002/cam4.2881

**Published:** 2020-01-29

**Authors:** Nadine Rohwer, Anja A. Kühl, Annika I. Ostermann, Nicole Marie Hartung, Nils Helge Schebb, Dieter Zopf, Fiona M. McDonald, Karsten‐H. Weylandt

**Affiliations:** ^1^ Medical Department Division of Hepatology and Gastroenterology Charite-Universitätsmedizin Berlin Berlin Germany; ^2^ Medical Department B Divisions of Hepatology, Gastroenterology, Oncology, Hematology, Rheumatology, Endocrinology and Diabetes Brandenburg Medical School Ruppin General Hospital Neuruppin Germany; ^3^ Department of Molecular Toxicology German Institute of Human Nutrition Potsdam-Rehbruecke Nuthetal Germany; ^4^ iPATH.Berlin–Immunopathology for Experimental Models Charité–Universitätsmedizin Berlin Berlin Germany; ^5^ Chair of Food Chemistry Faculty of Mathematics and Natural Sciences University of Wuppertal Wuppertal Germany; ^6^ Research and Development, Pharmaceuticals, Bayer AG Berlin Germany

**Keywords:** aspirin, colorectal cancer, cyclooxygenase, tumor prevention

## Abstract

Although early detection and treatment of colorectal cancer (CRC) have improved, it remains a significant health‐care problem with high morbidity and mortality. Data indicate that long‐term intake of low‐dose aspirin reduces the risk of CRC; however, the mechanisms underlying this chemopreventive effect are still unclear. Different mouse models for inflammation‐associated, sporadic, and hereditary CRC were applied to assess the efficacy and mechanism of low‐dose aspirin on tumor prevention. An initial dosing study performed in healthy mice indicates that aspirin at a dose of 25 mg/kg/d has a similar pharmacodynamic effect as low‐dose aspirin treatment in human subjects (100 mg/d). Chronic low‐dose aspirin treatment suppresses colitis‐associated and to a lesser extent spontaneous tumorigenesis in mice. Aspirin's antitumor effect is most pronounced in a preventive approach when aspirin administration starts before the tumor‐initiating genotoxic event and continues for the duration of the experiment. These effects are not associated with alterations in cell proliferation, apoptosis, or activation of signaling pathways involved in CRC. Aspirin‐induced reduction in tumor burden is accompanied by inhibition of thromboxane B_2_ formation, indicating reduced platelet activation. Aspirin treatment also results in decreased colonic prostaglandin E_2_ formation and tumor angiogenesis. With respect to colitis‐triggered tumorigenesis, aspirin administration is associated with a reduction in inflammatory activity in the colon, as indicated by decreased levels of pro‐inflammatory mediators, and tumor‐associated iNOS‐positive macrophages. Our results suggest that low‐dose aspirin represents an effective antitumor agent in the context of colon tumorigenesis primarily due to its well‐established cyclooxygenase inhibition effects.

## Graphical abstract

Using three different mouse models of intestinal tumorigenesis, this study clearly establishes a colon tumor‐protective effect of low‐dose aspirin. Aspirin‐induced reduction in tumor burden is accompanied by inhibition of TXB2 and PGE2 formation, as well as a decrease in tumor angiogenesis and inflammatory activity in the colon. These findings argue toward a classical prostanoid‐dependent antitumor effect of low‐dose aspirin.

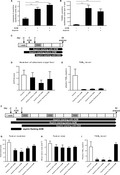

## 1 INTRODUCTION

Colorectal cancer (CRC) is one of the main health‐care challenges in the western world and accounts for approximately 10% of cancer‐related deaths.^1^ The pathogenesis of CRC is very complex and affected by multiple factors including genetic predisposition. Hereditary CRC accounts for about 5% of cases and the two most common syndromes are Lynch syndrome and familial adenomatous polyposis (FAP). Furthermore, it is well‐established that inflammatory bowel diseases are an important risk factor for the development of CRC.^2^ Many observations made in these special contexts of hereditary and inflammation‐triggered CRC have relevance also for the development of sporadic CRC. Despite advances in screening programs and medical and surgical therapies, cure rates and long‐term survival have improved only slightly over the past several decades and CRC still remains a huge problem with high morbidity and mortality.^3^

A considerable body of data from preclinical and clinical studies supports the hypothesis that the unselective cyclooxygenase (COX) inhibitor aspirin could be effective in the prevention of CRC.^4^, ^5^ Furthermore, there is also evidence for a possible role of aspirin in the treatment of CRC and the prevention of metastasis.^6^, ^7^, ^8^, ^9^, ^10^, ^11^ Although compelling evidence in humans demonstrates that low‐dose aspirin can prevent CRC, the molecular mechanisms are still uncertain. Generally, two different modes of chemoprevention can be distinguished: COX‐dependent and ‐independent mechanisms. Several COX‐independent mechanisms of aspirin have been suggested, for example, the modulation of the NF‐κB signaling pathway^12^, ^13^ and the inhibition of pro‐tumorigenic signaling pathways such as WNT/β‐catenin, ERK, and mTOR.^14^, ^15^, ^16^ However, alterations of COX‐related pathways are the primary focus of interest. Different from other nonsteroidal anti‐inflammatory drugs (NSAIDs), aspirin irreversibly inactivates both isoforms of the COX enzymes albeit with a higher affinity to COX‐1 than COX‐2. It is known that the inhibition of COX‐1 in platelets by low‐dose aspirin induces a permanent defect of platelet function. Interestingly, an increase in platelet activation has been shown in patients with CRC and platelet activation has been linked to key steps in cancer progression.^17^ However, one of the best recognized mechanisms of aspirin‐induced inhibition of COX enzymes is the decreased formation of pro‐tumorigenic eicosanoids such as prostaglandin E_2_ (PGE_2_). Generally, it is known that COX‐2 is transcriptionally upregulated in 80%‐90% of human colorectal carcinomas,^18^ resulting in enhanced prostaglandin production with PGE_2_ being the dominant product.^19^ Another unique property of aspirin, which is not shared by other NSAIDs is the acetylation of COX‐2. This acetylation can switch COX‐2 from synthesizing tumor‐promoting PGE_2_ to hydroperoxy‐ and hydroxy‐fatty acids which are precursors for anti‐inflammatory and pro‐resolution lipid mediators, such as 17(R)‐Resolvin D1 (17(R)‐RvD1) and 15(R)‐Lipoxin A_4_ (15‐R‐LXA_4_) which may contribute to the chemoprotective action of aspirin in CRC.^20^

In summary, published data establish a protective effect of low‐dose aspirin in the context of colon tumorigenesis. However, mechanistic evidence as to how this protection can be explained in vivo is still incomplete. Therefore, the aim of this study was to add mechanistic information to the clinically observed effectiveness of long‐term aspirin intake in CRC prevention with the use of three different mouse models of intestinal tumorigenesis: (a) the AOM/DSS model as a model for inflammation‐triggered colon cancer; (b) the AOM model as a model for sporadic colon tumor development; and (c) the APC^Min/+^ model which is considered to be a model for the hereditary FAP.

## 2 MATERIALS AND METHODS

### 2.1 Drug preparation and administration

Aspirin iv 500 mg (Bayer Vital GmbH) was dissolved in water and administered daily by oral gavage or in the drinking water. A daily water consumption of 0.15 L/kg body weight was used for dose calculation. For the mouse colon tumor models, an aspirin dose of 25 mg/kg/d was administered via drinking water. The animals in the control groups were mock‐treated by oral gavage or received normal drinking water.

### 2.2 Animals and mouse models

All animal experiments were conducted according to the European Guidelines for animal welfare with approval of the relevant regulatory agency (Landesamt für Gesundheit und Soziales Berlin, G0026/15).

For the AOM/DSS model, 6‐ to 8‐week‐old male and female C57BL/6J mice were injected intraperitoneally with 10 mg/kg AOM (azoxymethane; Sigma‐Aldrich) followed by three cycles of 2% dextran sodium sulfate (DSS; MP Biomedicals) in drinking water for 5 days and normal drinking water for 16 days. Mice were sacrificed 4 and 12 weeks after AOM injection. For tumor induction in the AOM model, 8‐ to 10‐week‐old female FVB/N mice were treated six times with 10 mg/kg AOM once a week by intraperitoneal injection and sacrificed 17 weeks after the first AOM administration. Tumors in the colon were counted and measured under a dissecting microscope and colonic tissue snap‐frozen. For tumor counting in the APC^Min/+^ model (C57BL/6J background), male and female mice were sacrificed at 100 days of age. In order to assess colitis activity, body weight, stool consistency, and the presence of occult or gross blood were determined and a scoring system was applied.^21^

### 2.3 Colon explant cultures and ELISA

Proximal colon segments were cultured for 24 hours in RPMI 1640 medium supplemented with 2% FCS (Biochrom), penicillin, streptomycin, and gentamycin (10 µg/mL, all from Gibco).

ELISA measurement of IL‐1β, IL‐6, TNFα, and VEGF‐A concentrations were performed on supernatants of colon explant cultures according to the manufacturer's instructions (eBioscience). Levels of TXB_2_ were quantified in the supernatants of colon explant cultures, plasma, or serum using ELISA kits according to the manufacturer's instructions (Cayman Chemical).

### 2.4 LC‐MS/MS analysis

PGE_2_, 17(R)‐RvD1, and 15(R)‐LXA_4_ were quantified in colon tissues by means of LC‐MS/MS as described before.^22^ The lower limits of quantification for 17(R)‐RvD1 and 15(R)‐LXA4 were 0.7 and 1 fmol/mg tissue, respectively.

### 2.5 RNA extraction and quantitative PCR analysis

Snap‐frozen colon tissue was pulverized with a TissueLyser II (Qiagen). Total RNA was isolated using the RNeasy Mini Kit (Qiagen) according to the manufacturer's instructions. Reverse transcription into cDNA was performed with the iScript Select cDNA Synthesis Kit (Bio‐Rad Laboratories). Quantitative real‐time PCR analysis was conducted on a CFX96 Real‐Time PCR Detection System using the SsoFast EvaGreen Supermix (both from Bio‐Rad Laboratories). Primer specificity was checked by melt‐curve analysis and DNA agarose gel electrophoresis of obtained PCR products. Relative fold changes of target gene expression were calculated by the comparative ΔCT method normalizing CT‐values to the geometric mean of the CT‐values of housekeeping genes. Primers sequences are listed in the Table S1.

### 2.6 Nuclear protein extraction and measurement of NF‐κB p65 activation

Nuclear extracts were prepared using a nuclear extract kit (Active Motif). To quantify NF‐κB p65 activation, the TransAM NF‐κB p65 activation assay (Active Motif) was used according to the manufacturer's instructions.

### 2.7 Immunohistochemistry

Immunohistochemical analysis was conducted on formalin‐fixed paraffin‐embedded tissues. Colon sections were stained with antibodies specific for β‐catenin (Cell Signaling Technology), BrdU (AbD Serotec), cleaved caspase‐3 (Cell Signaling Technology), CD31 (Dianova), c‐MYC (Santa Cruz Biotechnology), cyclin D1 (Santa Cruz Biotechnology), F4/80 (Life Technologies), iNOS (Abcam), and Ki‐67 (Dako Deutschland GmbH). The antibody‐antigen complexes were detected using biotinylated donkey anti‐rat and donkey anti‐rabbit secondary antibodies (Dianova) and the Dako REAL Detection System (Dako). Immunohistochemical detection of HIF‐1α (Cayman Chemical) was performed as previously described.^23^ Nuclei were counterstained with hematoxylin. Negative controls were performed by omitting the primary antibody. The average number of positively stained cells within at least six high power fields (HPF, 0.237 mm^2^) was determined by a blinded independent investigator.

### 2.8 TUNEL assay

Immunofluorescence detection of epithelial apoptosis was performed on colon sections with the TUNEL assay (Roche). The average number of positively stained epithelial cells within five high power fields (HPF, 0.287 mm^2^) was determined microscopically by an independent blinded investigator.

### 2.9 Statistical analysis

Statistical analysis was performed using GraphPad Prism software. Statistical significance was determined by two‐tailed Student's *t* test for unpaired observations. Differences were considered statistically significant at *P* < .05. Data are expressed as individual values±SEM or means+SEM.

## 3 RESULTS

### 3.1 Definition of an oral aspirin dose in mice that resembles low‐dose used in humans

The aim of this study was to investigate the tumor‐preventive effect of oral low‐dose aspirin on colon tumor development in mice. In humans, low‐dose aspirin is defined as the dose that leads to an antithrombotic action by irreversible inhibition of platelet COX‐1. There is general agreement that daily doses of 75‐100 mg aspirin are sufficient to inhibit plasma thromboxane B_2_ (TXB_2_) formation as a pharmacodynamic biomarker of COX‐1 inhibition in platelets. A 7‐day low‐dose aspirin administration (100 mg/d) to healthy human volunteers is associated with virtually complete suppression of platelet COX‐1 activity as reflected by reductions in serum TXB_2_ concentration by 95%‐99%.^24^ To identify an oral aspirin dose that resembles the human dose of 100 mg/d, mice were treated with aspirin for 7 days at 5 and 25 mg/kg/d by oral gavage or via drinking water. Our initial dosing study indicated that aspirin at a dose of 25 mg/kg/d had a similar pharmacodynamic effect to low‐dose aspirin treatment in human subjects and resulted in an inhibition of plasma and serum TXB_2_ formation by 85%‐95% at 3 hours after the last dose (Figures S1, S2; Table S2). Interestingly, the application of aspirin in the drinking water resulted in a reduction in plasma TXB_2_ levels comparable to that seen using application via oral gavage (Figure S1). Consistent with the results of the aforementioned clinical study,^24^ the inhibition of platelet TXB_2_ formation in mice remained stable up to 24 hours after the last dose of aspirin (Figure S1; Table S2). Accordingly, an aspirin dose of 25 mg/kg/d administered via drinking water was used for the experiments in the mouse colon tumor models.

### 3.2 Treatment with low‐dose aspirin suppresses colon tumor formation and ameliorates colonic inflammation in the AOM/DSS model

**Figure 1:**
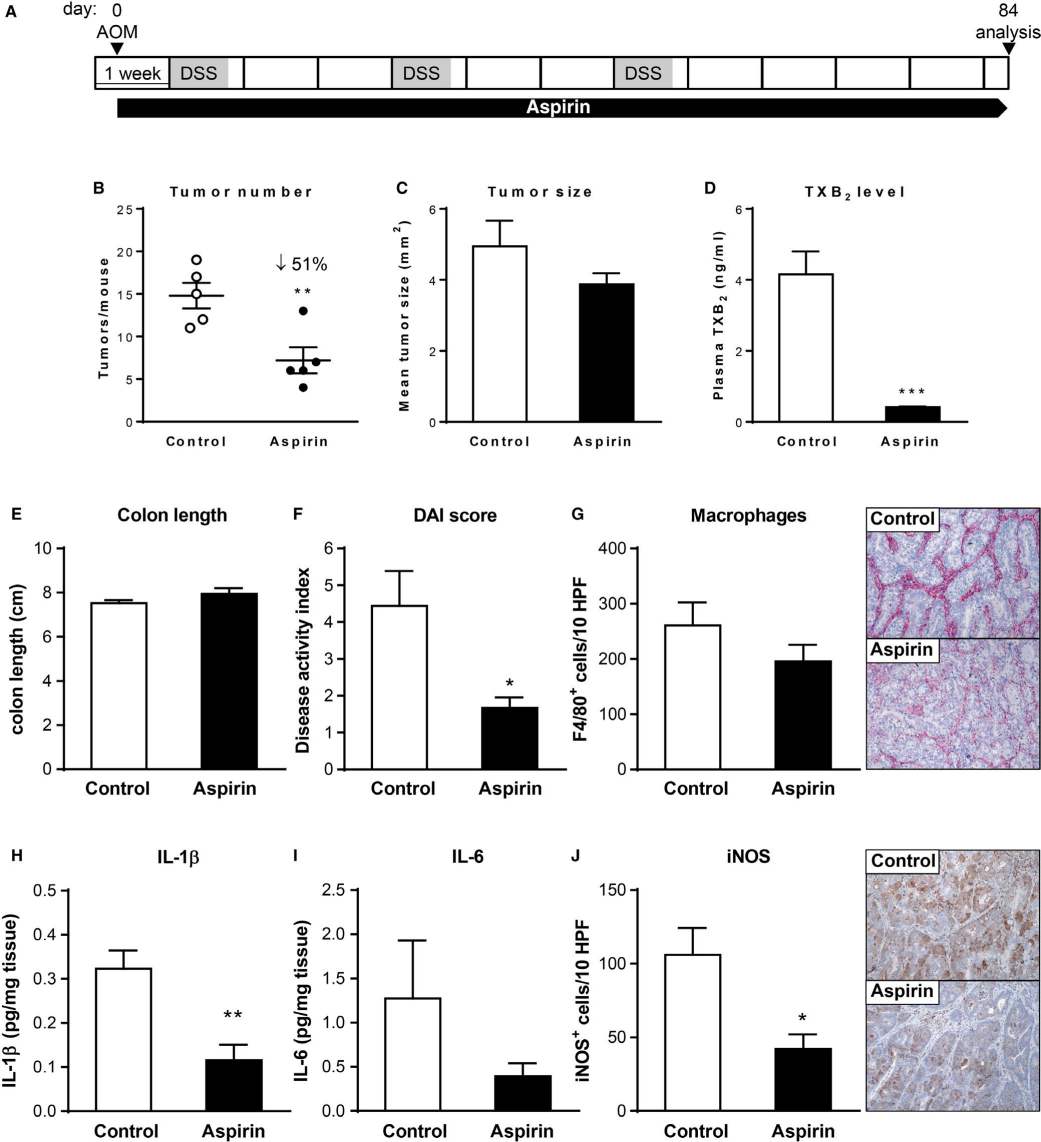
Effect of chronic low‐dose aspirin treatment on colon tumor formation and inflammation in the AOM/DSS model. (A) Schematic representation of the AOM/DSS and aspirin treatment protocol. (B) Tumor number, (C) tumor size; and (D) plasma TXB_2_ concentration of mice (n = 5/group) following 12 wks of aspirin administration. (E and F) Colon length (E, n = 5/group) and disease activity index (F, n = 9/group) of control and aspirin‐treated mice. (G and J) Representative images of immunohistochemical staining for the macrophage marker F4/80 (G) and the inflammatory marker iNOS (J) of colon sections (magnification ×100) and quantification of the number of positive cells from control and aspirin‐treated mice (n = 5/group). (H and I) IL‐1β (H) and IL‐6 (I) concentration in supernatants of colon explant cultures of control and aspirin‐treated mice (n = 5/group). *P < .05, **P < .01, ***P < .001 by unpaired Student's *t* test

In order to investigate the effect of low‐dose aspirin treatment in the setting of inflammation‐associated colon tumorigenesis, we used the AOM/DSS model (Figure 1A). Aspirin treatment was started concomitant with AOM administration and continued for 12 weeks in total (Figure 1A). At the endpoint of the experiment on day 84, we observed tumors in the distal and mid colon and rare occasional lesions in the proximal colon. Notably, aspirin significantly reduced the total tumor number per mouse by 51% compared to untreated control mice, whereas the decrease in the average size of individual tumors was less marked and not statistically significant (Figure 1B,C). Aspirin‐induced reduction in tumor burden was accompanied by reduction of systemic plasma TXB_2_ levels by 90% versus control, indicating reduced platelet activation (Figure 1D). TXB_2_ levels were also significantly reduced by 50% in supernatants from colon explants (Figure S3A).

Treatment with a higher dose of aspirin (50 mg/kg/d) resulted in a somewhat greater reduction in plasma TXB_2_ concentration compared to the 25 mg/kg/d dose (Figure S4C). However, increasing the aspirin dose to 50 mg/kg/d was not associated with an increase in antitumor efficacy (Figure S4A,B). The absence of increased antitumor efficacy with increased dose beyond 25 mg/kg/d indicates that—at least in this model—the tumor‐preventive effect of aspirin is indeed a low‐dose phenomenon associated with COX‐1 inhibition.

Recently, aspirin has been shown to exert protective effects during inflammation in mice and humans even at antithrombotic low doses.^25^, ^26^, ^27^ Therefore, we evaluated the effect of low‐dose aspirin treatment on the inflammatory response in the AOM/DSS model. Aspirin treatment was associated with an improvement in clinical signs of colitis, such as stool consistency and fecal bleeding, translating into a significantly reduced disease activity index of aspirin‐treated mice (Figure 1F). Furthermore, immunohistological analysis showed a trend toward reduced tumor infiltration by F4/80‐positive macrophages (Figure 1G) and significantly lower numbers of iNOS‐positive cells in colon sections of aspirin‐treated mice (Figure 1J). Consistently, the secretion of pro‐inflammatory cytokines, such as IL‐1β, by colon explants as well as mRNA levels of several pro‐inflammatory genes in the tumor tissue were significantly decreased following aspirin administration (Figure 1H; Figure S5).

In recent years, it has been shown that aspirin not only inhibits prostanoid biosynthesis but can, as a result of COX‐2 acetylation, lead to the formation of anti‐inflammatory, aspirin‐triggered lipid mediators, including 17(R)‐RvD1 and 15(R)‐LXA_4_. However, quantification by LC‐MS/MS showed that in this study both 17(R)‐RvD1 and 15(R)‐LXA4 concentrations were below the lower limit of quantification in colonic normal and tumor tissues of control and aspirin‐treated mice and prevented the assessment of an effect of aspirin on COX‐2.

Taken together, our data show that chronic low‐dose aspirin treatment significantly suppresses colon tumor development and ameliorates colonic inflammation in vivo in the inflammation‐triggered AOM/DSS model.

### 3.3 Low‐dose aspirin does not affect COX‐independent pathways of relevance in the AOM/DSS colon tumor model

**Figure 2:**
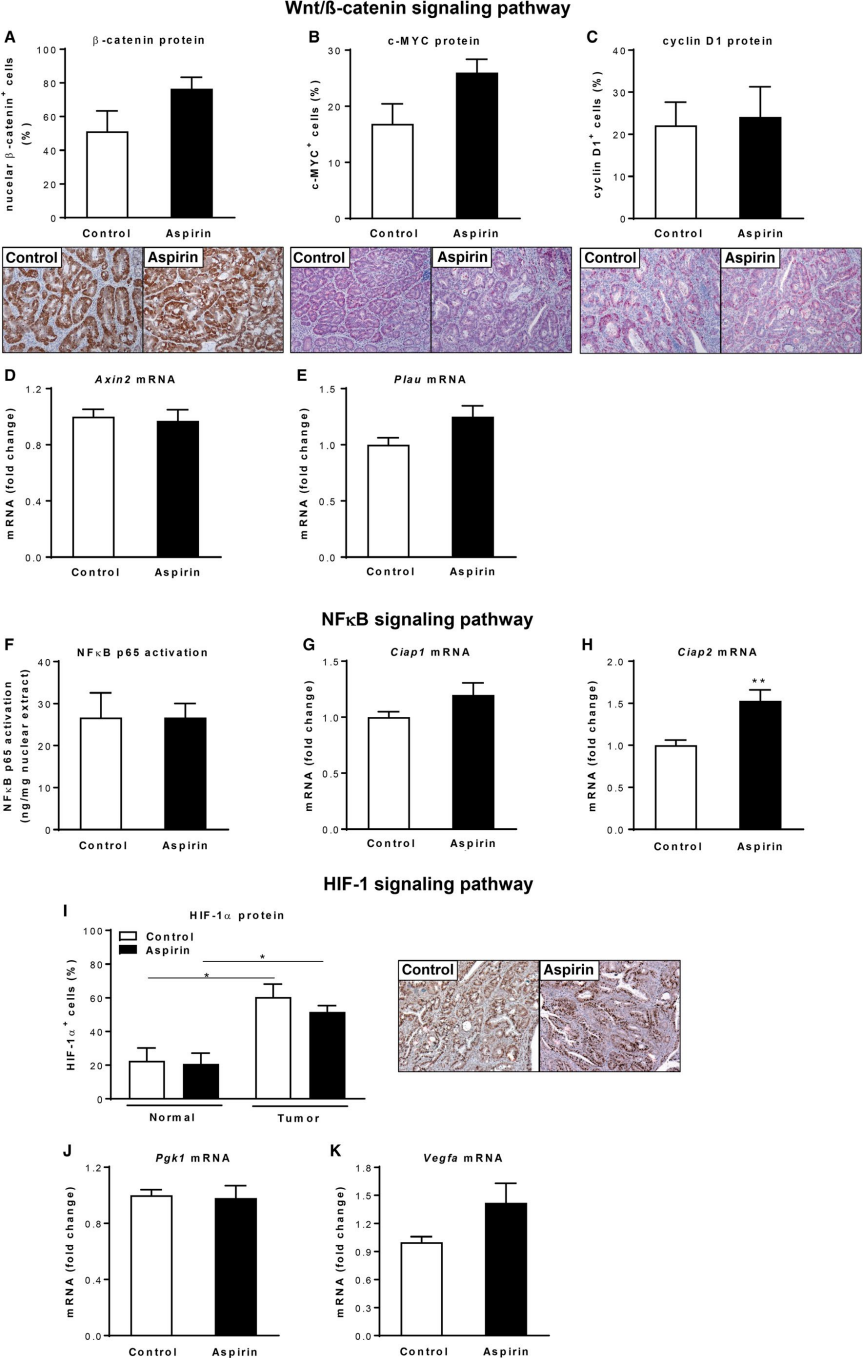
Analysis of signaling pathways involved in colon cancer following low‐dose aspirin treatment in the AOM/DSS model. (A‐C) Immunohistochemical analysis of β‐catenin (A) and wnt/β‐catenin targets c‐MYC (B) and cyclin D1 (C) staining of colons from control and aspirin‐treated mice (n = 4/group), magnification ×100. (D and E) Relative mRNA expression of wnt/β‐catenin target genes *Axin2* (D) and *Plau* (E) in colon tumors of control and aspirin‐treated mice (n = 5/group). (F) NFκB p65 activation in nuclear extracts from colon tumors (n = 3/group) after aspirin administration. (G and H) Relative mRNA expression of NFκB target genes *Ciap1* (G) and *Ciap2* (H) in colon tumors of control and aspirin‐treated mice (n = 5/group). (I) Immunohistochemical analysis of HIF‐1α staining of colon sections from control and aspirin‐treated mice (n = 3/group), magnification ×100. (J and K) Relative mRNA expression of HIF target genes *Pgk1* (J) and *Vegfa* (K) in colon tumors of control and aspirin‐treated mice (n = 5/group). *P < .05, **P < .01 by unpaired Student's *t* test

Recently, several COX‐independent modes of action of aspirin have been described that might contribute to its anticancer effects.^12^, ^13^, ^14^, ^15^, ^16^ Therefore, we next aimed to assess whether low‐dose aspirin treatment modulates two main pathways in CRC, namely the Wnt/β‐catenin and the NF‐κB signaling pathway in vivo in the AOM/DSS model. First, we analyzed the activation of the β‐catenin pathway by immunohistochemistry and qPCR. Immunohistochemical detection revealed that more than 50% of the tumor cells in the colon sections were positive for β‐catenin (Figure 2A). This was accompanied by nuclear accumulation of two β‐catenin target gene products c‐MYC and cyclin D1 (Figure 2B,C). However, no significant differences in numbers of β‐catenin‐, c‐MYC‐, or cyclin D1‐positive tumor cells between control and aspirin‐treated mice could be detected (Figure 2A,C). Similarly, tumor mRNA levels of β‐catenin target genes *Axin2* and *Plau* were not different between the control and aspirin groups (Figure 2D,E). Next, we determined NF‐κB p65 activation on nuclear extracts from colon tumors of control and aspirin‐treated mice. Activation of NF‐κB p65 in colon tumors was unaffected by aspirin treatment of mice (Figure 2F). Consistent with this finding, qPCR analysis revealed no reductions in tumor mRNA levels of NF‐κB target genes *Ciap1* and *Ciap2* by aspirin treatment (Figure 2G,H). Aspirin treatment thus did not result in significant modulations of the Wnt/β‐catenin or NF‐κB signaling pathway in vivo*.*

### 3.4 Aspirin's antitumor effect is most pronounced in the preventive setting

Given the significant reductions in tumor number but no apparent effect on tumor size in the AOM/DSS model, we concluded that aspirin treatment affects mainly early stages of tumor initiation and/or promotion. To dissect the order of intestinal processes and events affected by aspirin treatment, we analyzed early stages of the AOM/DSS regimen.

**Figure 3:**
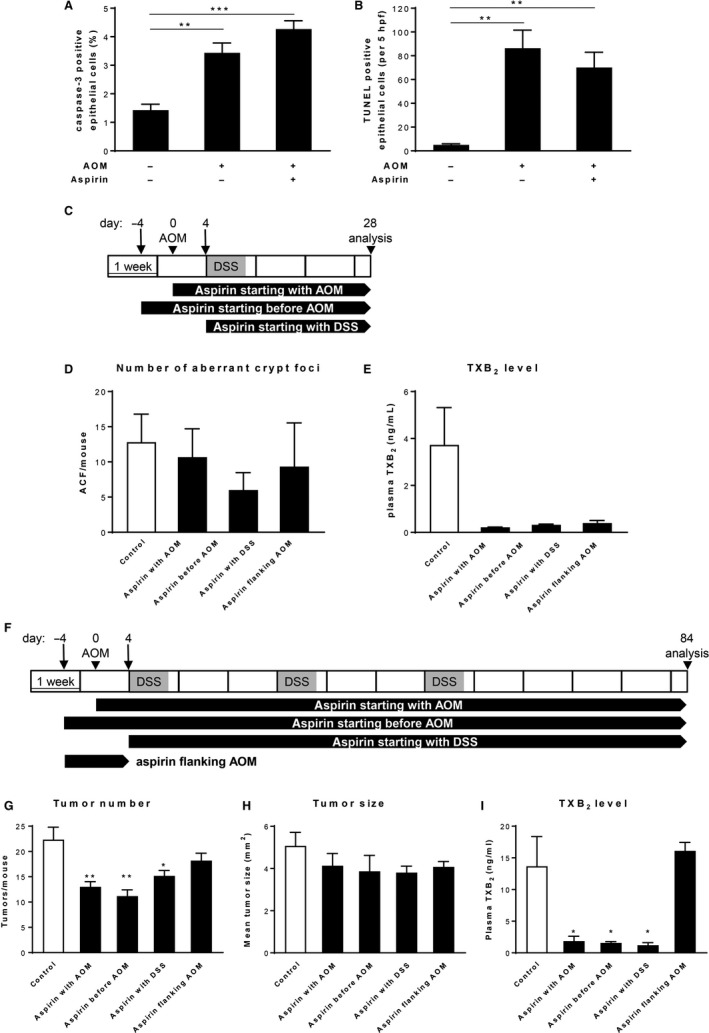
Effect of chronic low‐dose aspirin treatment on early stages in AOM/DSS‐induced tumorigenesis. (A and B) Assessment of apoptosis by cleaved caspase‐3 staining (A) and TUNEL assay (B) of colons from control and aspirin‐treated mice (n = 4‐6/group) 4 h after AOM administration. (C) Schematic representation of the AOM/DSS protocol and aspirin treatment regimens for assessment of aberrant crypt formation. (D) Number of aberrant crypt foci and (E) plasma TXB_2_ concentration of mice (n = 6/group) after 4 wks of aspirin administration. (F) Schematic representation of the 12‐week AOM/DSS protocol in combination with different aspirin treatment regimens. (G) Tumor number, (H) tumor size, and (I) plasma TXB_2_ concentration of mice (n = 6/group) following 12 wks of aspirin administration. *P < .05, **P < .01, ***P < .001 by unpaired Student's *t* test

First, we examined whether aspirin treatment renders enterocytes more susceptible to AOM‐induced apoptosis. We injected control and aspirin‐treated mice with AOM and sacrificed them 4 hours after AOM administration. As can be shown by both cleaved caspase‐3 staining and TUNEL assay, AOM administration to mice significantly induced apoptosis in epithelial colonic cells (Figure 3A,B). Aspirin treatment of AOM‐injected animals did not significantly change apoptosis observed by AOM administration alone (Figure 3A,B) which suggests that aspirin treatment did not influence tumor onset at this early time point of colon tumorigenesis by apoptosis induction. We did not check whether aspirin treatment enhances DNA repair to prevent mutagenesis induced by AOM. However, there is some evidence that aspirin treatment, at least in vitro, can result in an induction of DNA repair mechanisms.^28^, ^29^

Next, we aimed to analyze whether aspirin treatment modifies the very earliest steps in the transformation of normal epithelium to neoplasia. One of the earliest histopathological changes that can be seen in the colon before formation of colorectal polyps and that may lead to colon neoplasia are aberrant crypt foci (ACF). To induce ACF formation, C57BL/6J mice were treated with AOM plus one cycle of DSS and sacrificed 4 weeks after AOM administration (Figure 3C). Aspirin treatment was performed over the entire experiment and involved three different treatment arms: aspirin treatment starting 4 days before AOM administration, aspirin treatment starting along with AOM administration, and aspirin treatment starting concomitant with DSS application (Figure 3C). None of the applied aspirin treatment regimens resulted in a significant change in the number of ACF. However, ACF formation was somewhat more decreased when aspirin treatment was initiated before AOM administration (Figure 3D). Consistent with this, treatment initiation prior to AOM administration also resulted in the largest reduction in total tumor number after completion of a 12‐week AOM/DSS regimen (Figure 3F‐H). In contrast, aspirin administered only for 4 days prior until 4 days post AOM injection did not significantly reduce tumor burden (Figure 3G).

Collectively, our results demonstrated that aspirin's antitumor effect is most pronounced when treatment started before tumor initiation and continued for the duration of the AOM/DSS experiment.

### 3.5 Treatment with low‐dose aspirin also suppresses colon tumor formation in the AOM colon tumor model

**Figure 4:**
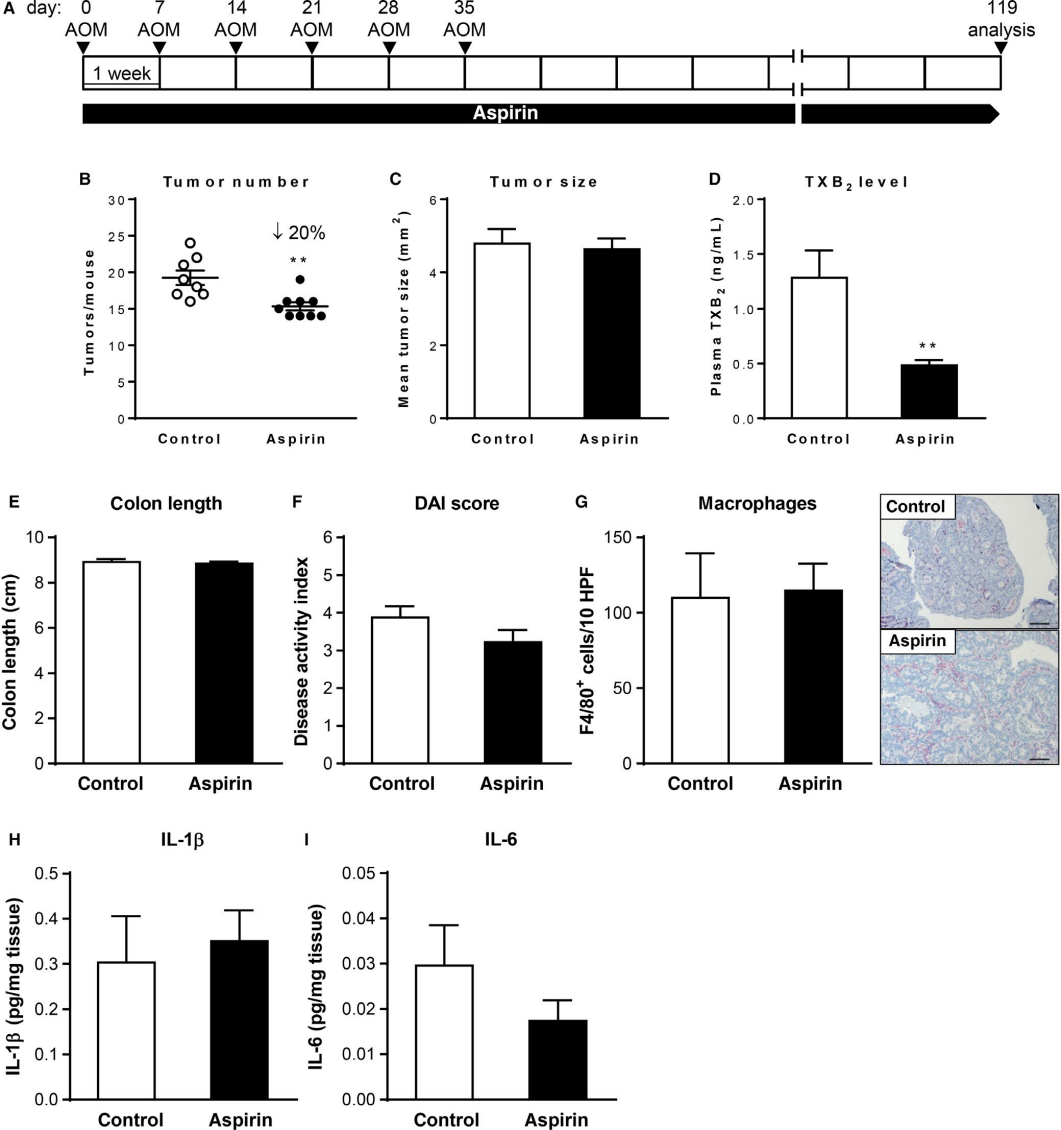
Effect of chronic low‐dose aspirin treatment on colon tumor formation and inflammation in the AOM model. (A) Schematic representation of the AOM and aspirin treatment protocol. (B) Tumor number, (C) tumor size, and (D) plasma TXB_2_ concentration of mice (n = 9/group) following 17 wks of aspirin administration. (E and F) Colon length (E) and disease activity index (F) of control and aspirin‐treated mice (n = 9/group). (G) Representative pictures of immunohistochemical staining for the macrophage marker F4/80 of colon sections (magnification ×100) and quantification of the number of positive cells from control and aspirin‐treated mice (n = 3‐4/group). (H and I) IL‐1β (H) and IL‐6 (I) concentration in supernatants from colon explant cultures of control and aspirin‐treated mice (n = 5/group). **P < .01 by unpaired Student's *t* test

Next, we aimed to address whether treatment with aspirin suppresses colorectal tumorigenesis also in the absence of chronic inflammation in the AOM model of sporadic colon tumor development. To this end, FVB/N mice were treated six times with AOM once a week (Figure 4A). Aspirin treatment started together with the first AOM administration and continued until the endpoint of the experiment on day 119. Efficacy of aspirin treatment was characterized by inhibition of TXB_2_ formation in both plasma and culture supernatants from colon explants and found to be highly efficient (Figure 4D; Figure S3B). All mice treated with AOM developed tumors that were mainly located in the middle to distal colon (data not shown). Aspirin‐treated mice showed a significant reduction in the total tumor number by 20% and no differences in the average tumor size when compared to control treated mice (Figure 4B,C).

Given the absence of inflammation at baseline in this model, low‐dose aspirin treatment had no significant effect on inflammatory disease activity index, cytokine production, or tumor‐associated macrophage numbers (Figure 4E‐I). In line with this, the number of F4/80‐positive macrophages was markedly lower and an iNOS expression not detectable in colon sections from AOM‐treated mice (Figure 4G) when compared to AOM/DSS‐treated mice (Figure 1G), reflecting the lower degree of inflammation in the AOM colon tumor model.

Taken together, this experiment shows that chronic low‐dose aspirin treatment also significantly suppresses colon tumor development but has no effect on colonic inflammation markers in the AOM model where tumor formation is not accelerated by an extrinsic inflammatory stimulus.

### 3.6 Low‐dose aspirin treatment inhibits tumor‐associated angiogenesis

**Figure 5:**
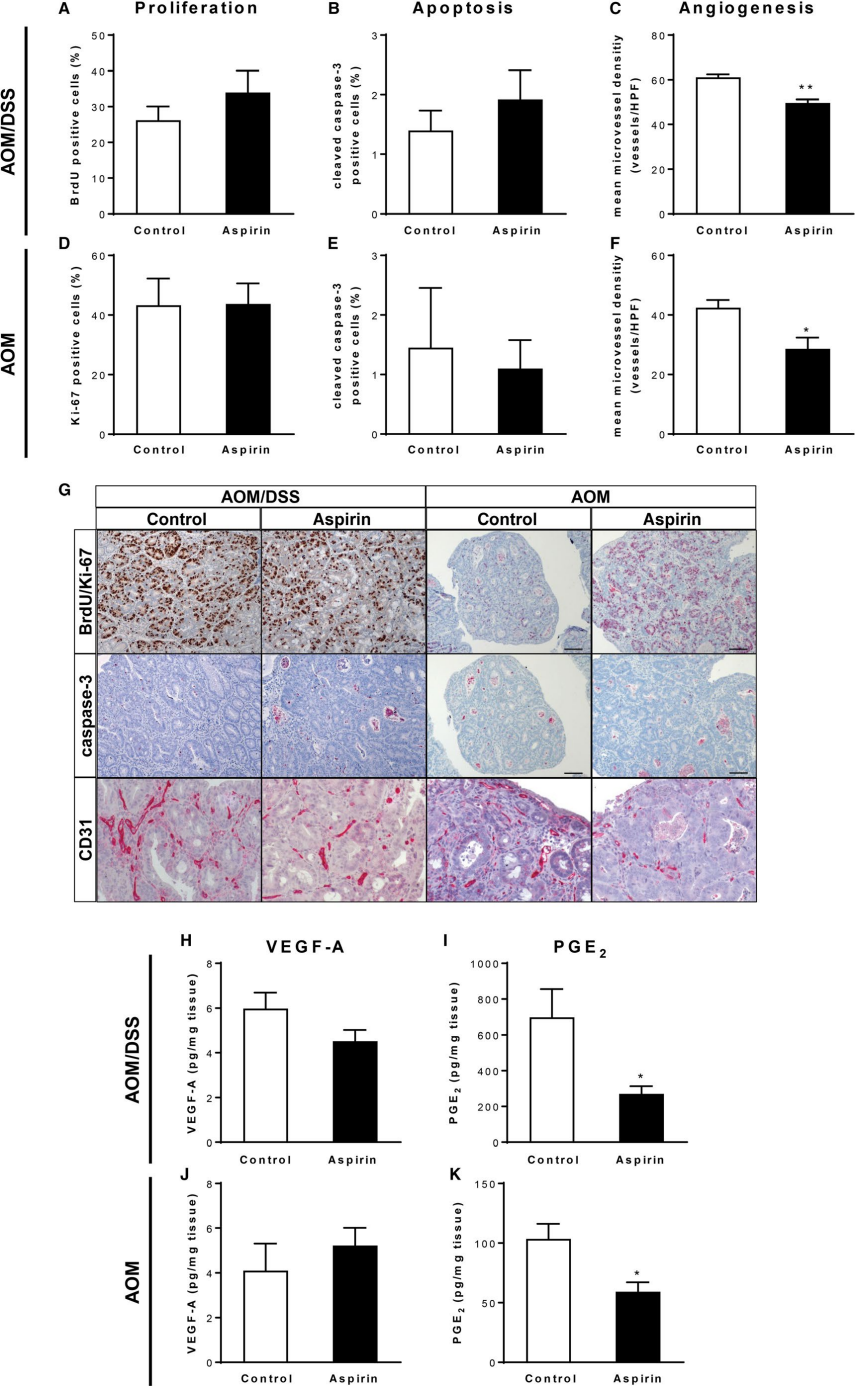
Assessment of basic malignant characteristics following low‐dose aspirin treatment in the AOM and AOM/DSS model. (A‐G) Immunohistochemical analysis of BrdU (A), Ki‐67 (D), cleaved caspase‐3 (B and E) and CD31 (C and F) staining of colons from control and aspirin‐treated mice (n = 3‐5/group) after completion of the AOM/DSS (A‐C) or AOM (D‐F) protocol. (G) Representative staining of colon sections from control and aspirin‐treated mice, magnification ×100. (H and J) VEGF‐A concentrations in supernatants from colon explants (n = 5‐9/group) after aspirin administration at the endpoint of the AOM/DSS (H) or AOM (J) protocol. (I and K) PGE_2_ concentrations in supernatants from colon explants (n = 5/group) after aspirin administration at the endpoint of the AOM/DSS (I) or AOM (K) protocol. *P < .05, **P < .01 by unpaired Student's *t* test

To further understand the mechanisms involved in the tumor‐preventive effect of aspirin, we analyzed basic malignant characteristics, such as abnormal proliferation, apoptosis evasion, and angiogenesis. To study epithelial cell proliferation, we performed BrdU or Ki‐67 staining. Quantification revealed no significant alterations in the proliferation rate between control and aspirin‐treated mice, neither in the AOM/DSS nor in the AOM model (Figure 5A,D,G). The apoptotic activity in tumor cells, measured by cleaved caspase‐3 staining, was also not significantly different between control and aspirin‐treated mice (Figure 5B,E,G). Interestingly, when assessing tumor angiogenesis by immunohistochemical detection of the endothelial marker CD31, the microvessel density was significantly reduced in tumors of aspirin‐treated mice in both colon tumor models (Figure 5C,F,G). Intrigued by this finding, we characterized the HIF‐1/VEGF pathway as one master regulator of angiogenesis in cancer. However, concentrations of VEGF‐A protein and mRNA were not significantly different between control and aspirin‐treated mice (Figure 5H,J; Figure 2K). In line with this observation, HIF‐1α protein abundance and tumor expression of the HIF‐1 target gene *Pgk1* in aspirin‐treated mice were not different from those in control mice (Figure 2I,J). Because it has been shown that aspirin decreases the formation of PGE_2_ in colorectal carcinomas and PGE_2_ can also promote tumor angiogenesis,^30^ we next measured PGE_2_ concentrations by LC‐MS/MS analysis. As expected, we found decreased secretion of PGE_2_ in colon culture supernatants of aspirin‐treated mice (Figure 5I,K).

In summary, these results show that the inhibition of colon tumor development in the AOM and AOM/DSS model by low‐dose aspirin treatment is accompanied by reductions in tumor‐associated angiogenesis which in turn could be due to decreased colon PGE_2_ formation.

### 3.7 Treatment with low‐dose aspirin does not significantly change colon tumor formation in the APC^Min/+^ model

**Figure 6:**
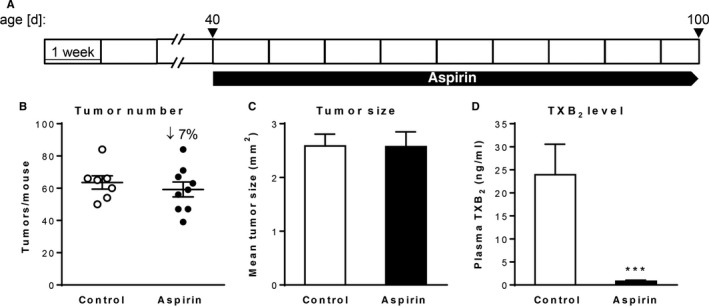
Effect of chronic low‐dose aspirin treatment on intestinal tumor formation in the APC^Min/+^ model. (A) Aspirin treatment schedule, (B) Tumor number, (C) tumor size, and (D) plasma TXB_2_ concentration of APC^Min/+^ mice (n = 7‐9/group) following 60 d of aspirin administration. ***P < .001 by unpaired Student's *t* test

Finally, we assessed the tumor‐preventive efficacy of aspirin treatment in APC^Min/+^ mice which carry a missense mutation in one allele of the tumor suppressor gene APC. In common with the human FAP syndrome, the APC^Min/+^ mouse model relies mainly on the deregulation of the Wnt/β‐catenin signaling pathway, therefore addressing another route of intestinal carcinogenesis. In light of our results in the AOM/DSS and AOM models, we expected even smaller aspirin effects on tumor formation in the APC^Min/+^ mouse model. We started aspirin treatment in APC^Min/+^ mice at 40 days of age and treatment was continued until 100 days of age (Figure 6A). Regardless of the treatment, all APC^Min/+^ mice developed multiple adenomas in the small intestine and rare large tumors in the colon. Indeed, as we expected from our previous experiments in other models, and as described in the literature before,^31^, ^32^, ^33^ low‐dose aspirin treatment of APC^Min/+^ mice did not result in a significant reduction in the total tumor number and the average tumor size compared to control APC^Min/+^ mice (Figure 6B,C). However, the efficacy of aspirin treatment was confirmed by a significant decrease of TXB_2_ production in plasma samples from aspirin‐treated APC^Min/+^ mice in comparison to control APC^Min/+^ mice (Figure 6D).

Using three different mouse models of CRC, we can thus conclude that chronic low‐dose aspirin treatment significantly suppresses colitis‐associated tumor development in the AOM/DSS model and to a lesser extent sporadic tumorigenesis in the AOM model most likely specifically due to its suppression of COX‐derived lipid mediators but has no significant effect on intestinal tumor formation in the APC^Min/+^ mouse model.

## 4 DISCUSSION

The unselective COX inhibitor aspirin has been used for more than 100 years at various dose levels for the treatment of a wide range of clinical symptoms and diseases. Based on its antiplatelet effects, oral low‐dose aspirin is also approved for the prevention of thrombotic cardiovascular diseases associated with platelet activation and aggregation. Epidemiological studies in patients prophylactically taking aspirin for cardiovascular disease prevention indicated also a beneficial effect of aspirin for the prevention of CRC. This was substantiated by multiple clinical studies including a meta‐analysis of four randomized controlled trials clearly showing that aspirin treatment for 5 or more years at doses as low as 75 mg/d reduced long‐term CRC risk by 24%.^34^, ^35^ Similarly, a pooled analysis of ten cohort and case‐control studies demonstrated that low‐dose aspirin use lowers the risk of CRC by 29%.^36^ Several preclinical studies on colon cancer cells and colon tumor models have also confirmed antineoplastic properties of aspirin. However, the evidence for the chemopreventive efficacy of aspirin in mouse models of CRC is ambiguous. In five published studies aspirin suppressed colon malignancy in mice,^37^, ^38^, ^39^, ^40^, ^41^ while in four others it failed to show efficacy.^31^, ^32^, ^33^, ^42^ This discrepancy is probably related to differences in the administration route, the aspirin dose, the mouse model which was used, and the treatment regimen in these studies. A lack of an antineoplastic effect was observed most often in studies using therapeutic treatment approaches and after dietary administration of aspirin.^31^, ^32^, ^42^ In the present study, aspirin was administered in the drinking water as a preventive treatment approach. Administration in the drinking water makes assessment of exact dose difficult per mouse, however, we tried to control for this by (a) comparing the COX‐1 inhibitory effect of the drinking water administration with gavaging at a dose of 25 mg/kg in initial pharmacodynamic dosing experiments (Figure S1; Table S2); and (b) by regular control of the consumed drinking water volume that mice achieved a similar dose. Moreover, aspirin administration via drinking water has been used in other murine disease models before.^43^, ^44^, ^45^, ^46^

Our results suggest that low‐dose aspirin represents an effective tumor‐preventive agent in the context of inflammation‐associated colon tumorigenesis. In the AOM model for sporadic colon tumor development, the aspirin‐induced reduction in tumor incidence was still significant, but with a less marked effect, while in the APC^Min/+^ model aspirin treatment failed to show significant tumor‐preventive efficacy. As the majority of adenomas in APC^Min/+^ mice are initiated in utero or shortly after birth, the aspirin treatment approach used by us for treatment of APC^Min/+^ mice corresponds more to a therapeutic rather than a preventive approach and probably explains the lack of an antitumor effect in this model.

The stronger antitumorigenic effect of aspirin seen in the AOM/DSS model compared to the AOM model can probably be explained by the additional anti‐inflammatory action of aspirin that we observed in the former model. A beneficial effect of aspirin on inflammation even at low antithrombotic doses has been shown before in humans and mice, for example, on inflammation in murine atherosclerosis and in a mouse model of active tuberculosis.^25^, ^26^, ^27^ Morris and colleagues found in humans that antithrombotic doses of aspirin dampen innate immune‐mediated responses by preventing polymorphonuclear leukocyte and macrophage accumulation in response to local tissue injury.^27^ They also provide evidence that low‐dose aspirin exerts its anti‐inflammatory effect by triggering formation of endothelial 15(R)‐LXA_4_ along with increased expression of its receptor ALX at the leukocyte/endothelial cell interface. 15(R)‐LXA_4_ was not detectable in colon tissues of aspirin‐treated mice by us and therefore our data do not support a role of this mechanism in the context of aspirin's antitumor effect in the colon. However, comparable to Morris et al we also observed a reduced infiltration of cells of the innate immune system (iNOS+ macrophages) in the inflammation‐triggered AOM/DSS model following aspirin treatment. Macrophages are one of the main infiltrating immune cells in chronic inflammation, secreting inflammatory factors and cytokines, which are components of essential oncogenic signaling pathways such as TNFα‐NFκB or the IL6‐STAT3. In line with this, the expression and/or secretion of pro‐tumorigenic cytokines, including TNFα, IL‐6, and IL‐1α, were significantly decreased in tumors of aspirin‐treated mice. In the current study, aspirin treatment did not lead to significant changes in NF‐κB signaling, while a regulation of the STAT3 pathway was not investigated. However, Tian et al demonstrated in vivo a reduction in STAT3 phosphorylation in mice with AOM/DSS‐induced colon tumors following aspirin treatment.^42^ Thus, low‐dose aspirin might lead, by a reduced secretion of pro‐inflammatory cytokines, to a modulation of the IL6‐STAT3 signaling pathway resulting in suppression of oncogenesis in the inflammation‐associated AOM/DSS model.

It is well known that platelets play key roles in tumor growth and metastasis and that in turn tumors, including CRC, can stimulate platelet activation.^17^ In the present study, we observed a significant and almost complete inhibition of plasma TXB_2_ production by 7 or more days of low‐dose aspirin administration, indicating reduced platelet activation in aspirin‐treated animals. Hence, the antiplatelet action of aspirin could be responsible, at least in part, for its antitumor effect in the current study. Recently, Guillem‐Llobat et al showed that low‐dose aspirin administration prevented the increase of the metastatic potential of the human colon carcinoma cell line HT29 in mice induced by in vitro exposure to human platelets.^47^ In line with these results, Lichtenberger and colleagues demonstrated that aspirin attenuates platelet‐induced proliferation, invasion, and epithelial‐mesenchymal transition of colon cancer cells in vitro and reduces the number of circulating platelets and platelet accumulation in the colon in the AOM/DSS model in vivo.^48^ Both studies provide evidence for anti‐metastatic properties of low‐dose aspirin, which is also supported by us. Chronic low‐dose aspirin treatment is accompanied by reductions in tumor‐associated angiogenesis in the AOM and AOM/DSS model. Angiogenesis is required for invasive tumor growth and metastasis, and tumor angiogenesis is thought to depend on a shift in the balance between pro‐ and anti‐angiogenic factors. In addition to our results, multiple studies have shown that aspirin treatment suppresses metastasis in different rodent in vivo models including an osteosarcoma xenograft model, a hepatocellular carcinoma model, and a lymph node metastasis model.^7^, ^8^, ^9^ Moreover, a meta‐analysis of five randomized controlled trials indicated that daily intake of low‐dose aspirin prevents distant metastasis in cancer patients. Several angiogenic factors involved in tumor‐dependent angiogenesis have been described, one of the main pro‐angiogenic factors is VEGF‐A. In the present study, *Vegfa* mRNA levels and VEGF‐A secretion in colon tissues were not significantly different between control and aspirin‐treated mice and therefore the anti‐angiogenic effect of chronic low‐dose aspirin seems to be independent of the VEGF pathway in the colon tumor models used by us. Recently, Etulain et al also showed that aspirin could block platelet‐induced angiogenesis in vitro independently of VEGF activity^49^ and Xu et al identified PGE_2_ as a soluble tumor‐derived angiogenic factor associated with VEGF‐independent angiogenesis in preclinical breast and colon cancer models.^50^ As we also observed significant reductions in colon PGE_2_ formation in aspirin‐treated animals, we speculate whether the aspirin‐induced suppression of tumor angiogenesis is attributed to the inhibition of the COX/PGE_2_ pathway in our models. The importance of COX‐2 and PGE_2_ in the pathophysiology of CRC is further strengthened by beneficial results that were also found for COX‐2 selective inhibitors in sporadic and hereditary CRC.^51^, ^52^ Considering the bipartite functions of PGE_2_ in inflammation and cancer,^53^ the role of PGE_2_ in inflammation‐associated colon cancer is not fully understood. The role of PGE_2_ in inflammation is multifaceted and both cell type and context specific. In the context of acute inflammation, the pro‐inflammatory effects of PGE_2_ are well‐established. However, PGE_2_ also elicits immunosuppressive properties that lead to resolution of inflammation and subsequent tissue repair. Within the context of cancer, PGE_2_ is considered to possess potent tumor‐promoting activity by inhibition of apoptosis and stimulation of epithelial cell proliferation, motility, and angiogenesis. Moreover, PGE_2_ also suppresses the antitumor immune response by triggering the activation and expansion of regulatory T cells and myeloid‐derived suppressor cells, and by impairment of dendritic cell function.^54^ Given the higher degree of inflammation and the about 6‐fold higher PGE_2_ levels in tumor tissues of control mice in the AOM/DSS model, the PGE_2_‐mediated suppression of the antitumor immunity possibly plays a more important role here and the aspirin‐induced reduction of PGE_2_ results in the observed stronger antitumor effects compared to the AOM model.

Apart from the COX‐1– and COX‐2–related pathways, several COX‐independent mechanisms of aspirin have been reported that might contribute to its anticancer effects. In the current study, aspirin treatment did not result in significant modulations of the Wnt/β‐catenin and NF‐κB signaling pathway reported to be regulated by aspirin in a COX‐independent way. However, most of the aspirin‐induced COX‐independent effects have been found in vitro and often require high aspirin concentrations, which might not be obtainable in the systemic circulation after treatment with low‐dose aspirin.

In summary, we used three different CRC mouse models to demonstrate the preventive effect of the chronic use of low‐dose aspirin. Our study suggests that low‐dose aspirin represents an effective antitumor agent especially in the context of inflammation‐associated colon tumorigenesis. The effect of aspirin on intestinal inflammation and platelet activation as well as on tumor angiogenesis might all contribute to the beneficial effects seen mainly in the inflammation‐triggered colon tumor model. However, in this study, we did not find evidence for a biological role of apoptosis, modulation of COX‐independent signaling pathways or formation of aspirin‐triggered lipid mediators by acetylated COX‐2. The effects of aspirin on colon inflammation and tumor angiogenesis observed by us could be a consequence of COX‐1 inhibition and argue for a classical prostanoid‐dependent antitumor effect of low‐dose aspirin in the experimental settings used by us. We believe this to be plausible, given that prostanoids were—in addition to their pro‐inflammatory role—shown to have pro‐tumorigenic effects as outlined above. However, this might be different in human colon carcinogenesis or in the context of other models or tumor entities, in which other mechanisms might have a role as well.

Finally, we demonstrated that aspirin's antitumor effect is most pronounced when treatment is started before tumor initiation and continued for the duration of the experiment. Hence, the results of our preclinical study are in line with the recommendations of the US Preventive Services Task Force for primary prevention of CRC in human subjects to use aspirin prophylactically and for a longer period (at least 10 years).^55^

## CONFLICT OF INTEREST

This preclinical study was supported by Bayer AG (Nadine Rohwer and Karsten‐H. Weylandt). Dieter Zopf and Fiona M. McDonald are full‐time employees of Bayer AG. The remaining authors disclose no conflicts.

## AUTHOR CONTRIBUTIONS

Nadine Rohwer, Dieter Zopf, Fiona M. McDonald; and Karsten‐H. Weylandt conceptualized and designed the study; Nadine Rohwer, Anja A. Kühl, Nicole Marie Hartung and Annika I. Ostermann acquired the data; Nadine Rohwer, Nils H. Schebb, Fiona M. McDonald, and Karsten‐H. Weylandt analyzed and interpreted the data; Nadine Rohwer and Karsten‐H. Weylandt drafted the manuscript; Nils H. Schebb, Dieter Zopf, Fiona M. McDonald, and Karsten‐H. Weylandt critically revised the manuscript and for important intellectual content; Nadine Rohwer statistically analyzed the data; Anja A. Kühl, Nicole Marie Hartung, Annika Ostermann, and Nils H. Schebb provided technical or material support; Karsten‐H. Weylandt supervised the study.

## Data Availability

The data that support the findings of this study are available from the corresponding author upon reasonable request.
