# Supplementary material for: Effects of chronic low‐dose aspirin treatment on tumor prevention in three mouse models of intestinal tumorigenesis
Source: Cancer Med. 2020 Jan 29;9(7):2535–50. doi: 10.1002/cam4.2881 (PMC7131863; doi:10.1002/cam4.2881)
Supplement: Supplementary file 1 [file CAM4-9-2535-s001.pdf]

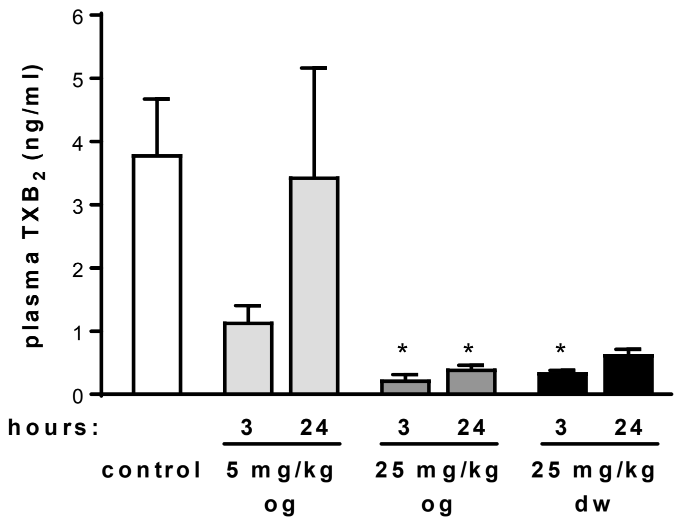

Figure S1. Plasma concentration of TXB_2 in healthy mice after aspirin administration. Mice (n = 3) were treated daily for 7 days with 5 or 25 mg/kg body weight of aspirin by oral gavage (og) or via drinking water (dw). Blood samples were collected 3 and 24 hours after the last aspirin dose. *P < .05 by unpaired Student's *t* test.

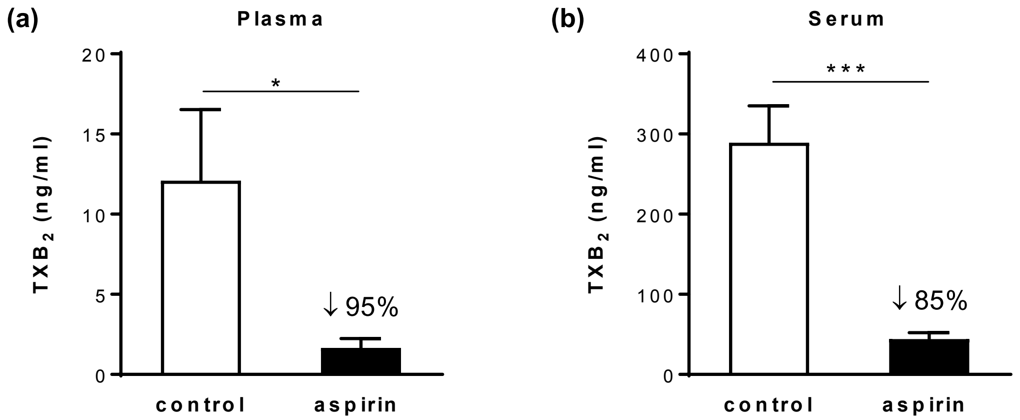

Figure S2. Plasma (*a*) and serum (*b*) concentration of TXB_2 in healthy mice after aspirin administration. Mice (n = 6) were treated daily for 7 days with 25 mg/kg body weight of aspirin via drinking water. Blood samples were collected 3 hours after the last aspirin dose. $^*P < .05$, $^{***}P < .001$ by unpaired Student's *t* test.

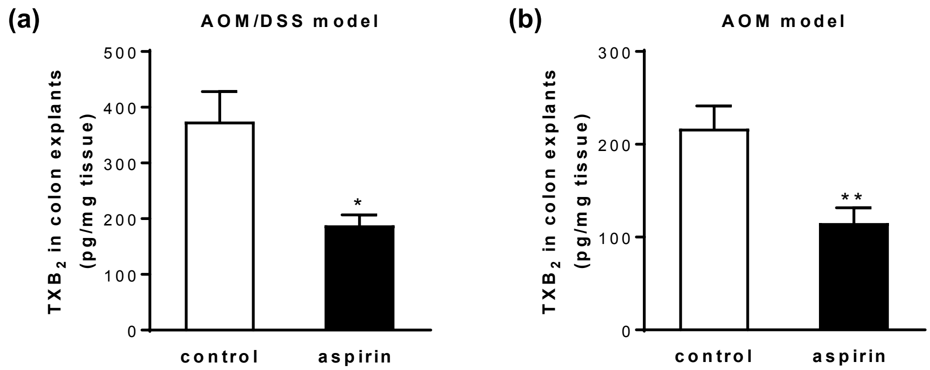

Figure S3. TXB_2 level in AOM/DSS- (*a*) and AOM-induced (*b*) murine colon tumors treated with aspirin. TXB_2 concentrations in culture supernatants from colon explants of aspirin-treated mice (25 mg/kg/day via drinking water) at the end of the AOM/DSS (n = 5/group, *a*) or AOM (n = 9/group, *b*) study. **P* < .05, ***P* < .01 by unpaired Student's *t* test.

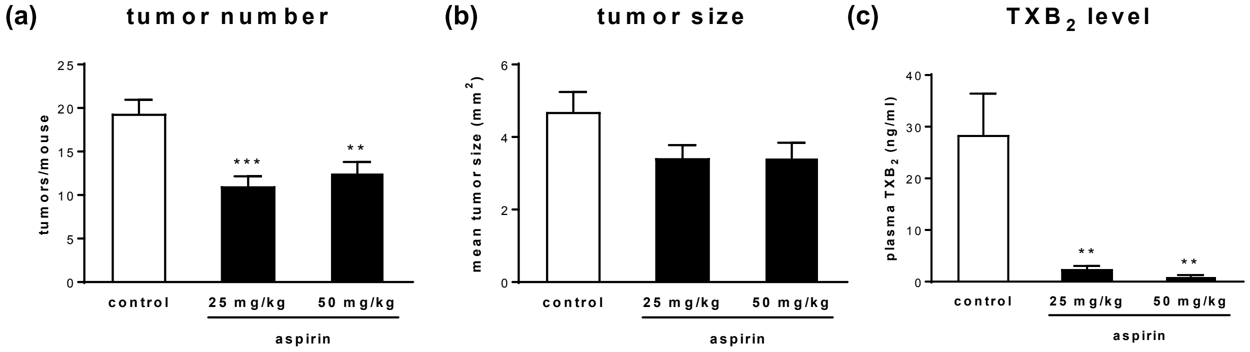

Figure S4. Aspirin dose dependency in the AOM/DSS model. (*a*) Tumor number, (*b*) tumor size and (*c*) plasma TXB_2 concentration of mice (n = 9/group) after 12 weeks of aspirin (25 or 50 mg/kg/day, via drinking water) administration in the AOM/DSS model. $^{**}P < .01$, $^{***}P < .001$ by unpaired Student's *t* test.

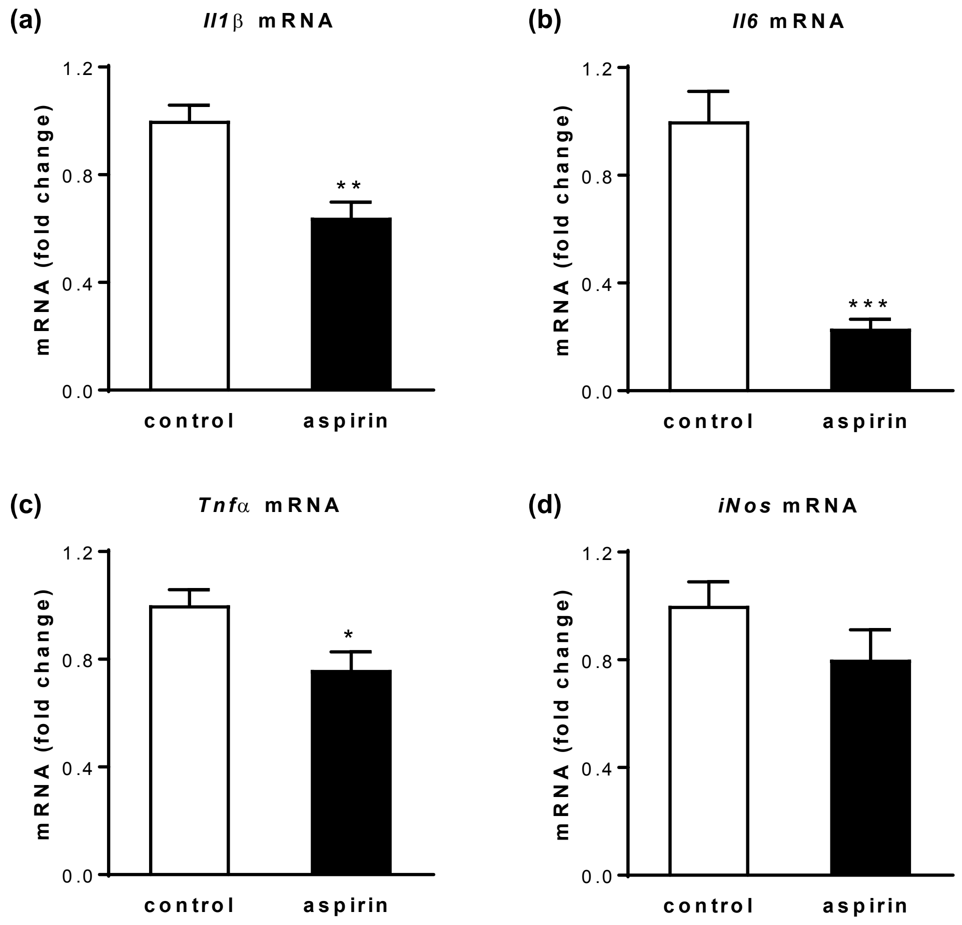

Figure S5. Quantitative qPCR analysis of pro-inflammatory genes. Relative mRNA expression of pro-inflammatory genes *Il1β* (*a*), *Il6* (*b*), *Tnfα (c)* and *iNos* (*d*) in colon tumors of control and aspirin-treated (25 mg/kg/day) mice (n = 5/group) following the AOM/DSS protocol. *$P < .05$, **$P < .01$, ***$P < .001$ by unpaired Student's *t* test.
