# Supplementary material for: Effects of chronic low‐dose aspirin treatment on tumor prevention in three mouse models of intestinal tumorigenesis
Source: Cancer Med. 2020 Jan 29;9(7):2535–50. doi: 10.1002/cam4.2881 (PMC7131863; doi:10.1002/cam4.2881)
Supplement: Supplementary file 2 [file CAM4-9-2535-s002.pdf]

Table S1. Murine primer sequences

Primer	Sequence
Axin2 forward primer	TTGACTGGGTCGCTTCTCTT
Axin2 reverse primer	GGGGGAAAACACAGCTTACA
Ciap1 forward primer	GGAAATTGACCCTGCGTTATACAGA
Ciap1 reverse primer	TCTCGGTCCATACACACTTTACACATT
Ciap2 forward primer	GGAAATTGACTCCACGTTATATGAAAACT
Ciap2 reverse primer	GCTCTTCCAATGACAAGCCTGA
Eef2 forward primer*	CTGCCTGTCAATGAGTCC
Eef2 reverse primer*	AGTATCAGGCTGCCTACAGT
Hprt1 forward primer*	TGGATACAGGCCAGACTTTGTT
Hprt1 reverse primer*	CAGATTCAACTTGCGCTCATC
Il-1β forward primer	CAACCAACAAGTGATATTCTCCATG
Il-1β reverse primer	GATCCACACTCTCCAGCTGCA
Il-6 forward primer	TGAGAAAAGAGTTGTGCAATGGC
Il-6 reverse primer	GCATCCATCATTTCTTTGTATCTCTGG
iNos forward primer	ACCCTAAGAGTCACCAAAATGGC
iNos reverse primer	TTGATCCTCACATACTGTGGACG
Pgk1 forward primer	CAAATTTGATGAGAATGCCAAGACT
Pgk1 reverse primer	TTCTTGCTGCTCTCAGTACCACA
Plau forward primer	GCCTGCTGTCCTTCAGAAAC
Plau reverse primer	TAGAGCCTTCTGGCCACACT
Rpl13a forward primer*	GCTTACCTGGGGCGTCTG
Rpl13a reverse primer*	ACATTCTTTTCTGCCTGTTTCC
Tnfa forward primer	CCATTCCTGAGTTCTGCAAAGG
Tnfa reverse primer	AGGTAGGAAGGCCTGAGATCTTATC
Vegfa forward primer	AGTCCCATGAAGTGATCAAGTTCA
Vegfa reverse primer	ATCCGCATGATCTGCATGG

* primer sequence of housekeeping gene

Table S2. Reduction of plasma TXB_2 level by aspirin treatment (% of the untreated control group)

**Time after last aspirin dose**	**3 hours**	**24 hours**
**5 mg/kg og**	69.77 ± 9.61	9.34 ± 49.66
**25 mg/kg og**	93.75 ± 2.41	89.34 ± 2.83
**25 mg/kg dw**	90.67 ± 2.22	83.09 ± 4.30
